# Ultrafast hydrogen bond dynamics of liquid water revealed by terahertz-induced transient birefringence

**DOI:** 10.1038/s41377-020-00370-z

**Published:** 2020-08-04

**Authors:** Hang Zhao, Yong Tan, Liangliang Zhang, Rui Zhang, Mostafa Shalaby, Cunlin Zhang, Yuejin Zhao, Xi-Cheng Zhang

**Affiliations:** 1 Beijing Key Laboratory for Precision Optoelectronic Measurement Instrument and Technology, School of Optics and Photonics, Beijing Institute of Technology, Beijing, 100081 China; 2 Beijing Advanced Innovation Center for Imaging Technology and Key Laboratory of Terahertz Optoelectronics (MoE), Department of Physics, Capital Normal University, Beijing, 100048 China; 3 Shenzhen Institutes of Advanced Technology, Chinese Academy of Sciences, Shenzhen, 518055 China; 4 The Institute of Optics, University of Rochester, Rochester, NY 14627 USA

**Keywords:** Terahertz optics, Nonlinear optics

## Abstract

The fundamental properties of water molecules, such as their molecular polarizability, have not yet been clarified. The hydrogen bond network is generally considered to play an important role in the thermodynamic properties of water. The terahertz (THz) Kerr effect technique, as a novel tool, is expected to be useful in exploring the low-frequency molecular dynamics of liquid water. Here, we use an intense and ultrabroadband THz pulse (peak electric field strength of 14.9 MV/cm, centre frequency of 3.9 THz, and bandwidth of 1–10 THz) to resonantly excite intermolecular modes of liquid water. Bipolar THz field-induced transient birefringence signals are observed in a free-flowing water film. We propose a hydrogen bond harmonic oscillator model associated with the dielectric susceptibility and combine it with the Lorentz dynamic equation to investigate the intermolecular structure and dynamics of liquid water. We mainly decompose the bipolar signals into a positive signal caused by hydrogen bond stretching vibration and a negative signal caused by hydrogen bond bending vibration, indicating that the polarizability perturbation of water presents competing contributions under bending and stretching conditions. A Kerr coefficient equation related to the intermolecular modes of water is established. The ultrafast intermolecular hydrogen bond dynamics of water revealed by an ultrabroadband THz pump pulse can provide further insights into the transient structure of liquid water corresponding to the pertinent modes.

## Hydrogen bond dynamics: Excitations for pickin’ up good vibrations

A new technique uses terahertz electric pulses to detect the stretching and bending vibrations in the bonds between water molecules. Liangliang Zhang of China’s Capital Normal University and colleagues found that applying a range of terahertz electric pulses on a film of free-falling water elicits measurable changes in refractive index that correspond to ultrafast hydrogen bond dynamics in liquid water. The pulses range from 1 to 10 terahertz, with the lower frequencies eliciting hydrogen bond bending vibrations and the higher frequencies eliciting stretching vibration signals. The approach enhances the relatively weak intermolecular bond vibrations, allowing scientists to distinguish them from other types of molecular motions. The method could be applied to study the physical mechanisms in water’s gas and crystalline phases, as well as the complex interactions between solvent water molecules and reagents.

## Introduction

Liquid water is considered the cornerstone of life and has many extraordinary physical and biochemical properties^1,2^. The hydrogen bond network of liquid water is widely recognized to play a crucial role in these properties^3–11^. The dielectric susceptibility *χ*(*ω*) reflects the collective and/or cooperative molecular motions with frequencies from the gigahertz (GHz) to terahertz (THz) range and is strongly influenced by the dynamics of intermolecular modes^12^. *χ*(*ω*) can be evaluated by various techniques, such as far-infrared spectroscopy^13,14^, low-frequency Raman spectroscopy^15,16^, THz time-domain spectroscopy^17,18^ and 2D Raman-THz spectroscopy^19^, which provide insights into the structure, stability and rearrangement dynamics of the hydrogen bond network. However, the determination of the molecular motions that make the dominant contributions to *χ*(*ω*) is difficult because the complexity of intermolecular interactions and the large spectral overlap of relevant modes, such as relaxation processes and intermolecular modes, make the assignment of the underlying dynamics challenging.

The time-resolved optical Kerr effect (OKE) technique^20–23^ is a powerful experimental tool enabling accurate investigation of dynamic phenomena in molecular liquids. However, since the rotation of a water molecule’s permanent dipole moment and other low-frequency motion modes cannot follow the ultrafast electric field oscillation, the contribution of water molecular motions to *χ*(*ω*) is much smaller than that of electron cloud distortion. Therefore, when the OKE technique is used to investigate intermolecular hydrogen bond motions, the birefringence signal is always positive, and the molecular response is extremely weak. An enhanced molecular response can be obtained when using a THz electric field, as the field is in resonance with the rotational transitions of a single molecule or the cooperative low-frequency molecular motions^24–26^. Therefore, the time-resolved THz Kerr effect (TKE) response is expected to be a powerful phenomenon for exploring the low-frequency dynamics of water molecules.

Recently, researchers have successfully used THz pulses to drive liquids, such as dimethyl sulfoxide (DMSO), chloroform and liquid water, and tracked the molecular dynamics by detecting transient birefringence^24,27–33^. In particular, in 2018, Zalden et al. used a low-frequency single-cycle THz pulse (centre frequency of 0.25 THz) generated by optical rectification in a LiNbO_3_ crystal to stimulate liquid water and observed the time-resolved birefringent signal induced by the transient orientation of dipole moments. They concluded that the polarizability of a water molecule parallel to the direction of the permanent dipole moment is smaller than the polarizability perpendicular to the permanent dipole moment^32^. However, only the polarizability anisotropy caused by the orientational relaxation of liquid water was investigated in this study, which is limited by the relatively low-power and narrowband properties of the applied THz excitation. In the latest research, Novelli et al. presented a narrowband THz pump/THz probe experiment using a free electron laser to induce strong anisotropy of liquid water through librational excitation^33^. A very large third-order response of liquid water at 12.3 THz was observed, which was three to five orders of magnitude larger than the previously reported values. However, their experiment using an individual probe pulse with a 3.6 ps full width at half maximum (FWHM) did not present the time-resolved measurement result. From this literature survey, it is concluded that for the time-resolved transient evolution of water molecular dynamics, only the orientation process excited by low-frequency THz waves is currently observed. The significant intermolecular modes of liquid water, such as the intermolecular hydrogen bond bending vibration at ~1.8 THz (i.e., 60 cm^−1^) and intermolecular hydrogen bond stretching vibration at ~5.7 THz (i.e., 190 cm^−1^)^15,16,18,19,31,34^, have not yet been investigated. As the absorption coefficients of water at the frequencies of the intermolecular modes (*α* = 330.18 cm^−1^ at 1.8 THz and *α* = 1201.0 cm^−1^ at 5.7 THz) are much larger than that at the frequency of the lower-frequency relaxation mode (*α* = 118.01 cm^−1^ at 0.25 THz)^35,36^, the application of the TKE technique in hydrogen bond dynamic research has remained challenging. Furthermore, all of the previous studies adopted cuvettes as liquid containers, which make the extraction of effective information from measurements difficult since the complex molecular interaction force with water and large Kerr response of a cuvette overwhelm the water response^18,29,32^.

Here, an ultrabroadband (centre frequency of 3.9 THz) and intense (peak electric field strength of 14.9 MV/cm) THz pulse is utilized to excite a gravity-driven, free-flowing water film. The THz pulse ranges from 1 to 10 THz and covers the two modes of intermolecular motion in the dielectric spectrum of water, which are hydrogen bond bending and stretching vibrations. For the first time, a bipolar TKE signal of liquid water with significant oscillation characteristics is observed. We propose a hydrogen bond harmonic oscillator model to explain the microscopic intermolecular motions. Moreover, we attribute the positive polarity to the intermolecular hydrogen bond stretching vibration and the negative polarity to the hydrogen bond bending vibration. Our theoretical analysis successfully assigns the potential molecular motions and agrees well with the experimental results. Our results provide a more intuitive time-resolved evolution of the polarizability anisotropy induced by the dynamics of the collective intermolecular modes of liquid water on the sub-picosecond scale.

## Results

### The Kerr effect in liquid water

**Fig. 1:**
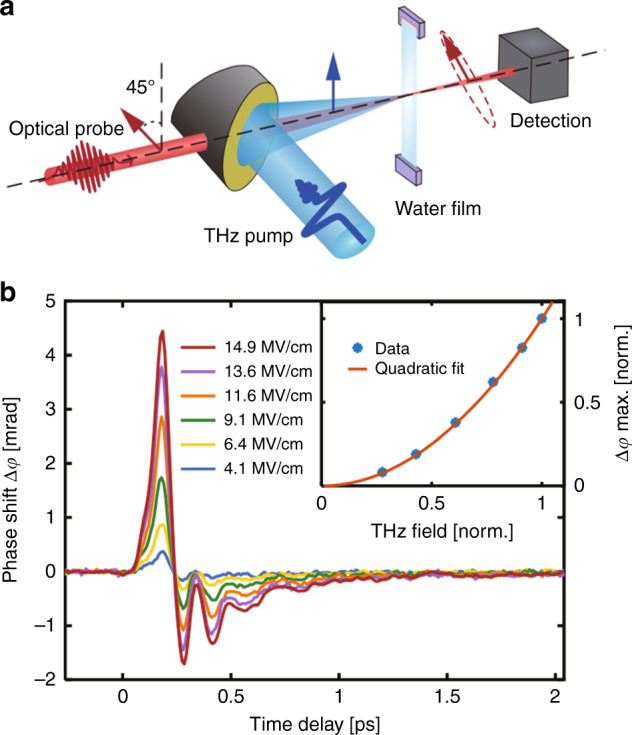
TKE measurement of liquid water. **a** Schematic diagram of the experimental system. A broadband THz pump pulse excites liquid water to initiate transient birefringence, which is monitored by an 800nm probe pulse that becomes elliptically polarized as it passes through the water film. **b** THz-induced Kerr effects in water with THz pump pulses of different peak electric field strengths. The inset shows the THz electric field strength dependence of the TKE response

A schematic diagram of the experimental system is shown in Fig. 1a. We use an ultrabroadband THz pulse with a centre frequency of 3.9 THz to excite the polarizability anisotropy of liquid water (see the “Materials and methods” section and Supplementary Information for details). A gravity-driven flowing water film instead of a traditional cuvette is applied to achieve a high signal-to-noise ratio and improve the accuracy of the experimental data, which is more convincing for analysing the physical characteristics of liquid water. The resulting transient birefringence is monitored by time-delayed optical probe pulses. As shown in Fig. 1b, we obtain a set of bipolar signals with clear oscillatory characteristics under different THz electric field strengths. We normalize all the curves (see the Supplementary Information for details) and find that all features of the responses scale with the square of the THz electric field, indicating that the Kerr effect is dominant during this process.

Compared with the negative polarity signal with an obvious relaxation tail extending over a few picoseconds in a previous study^32^, our birefringent signals should have different causes than the molecular orientational relaxation of liquid water. Taking into account the dielectric spectrum of water^15,16,18,19,31,34^, the THz band in our work mainly covers the two modes of intermolecular hydrogen bond motion, which indicates that the transient birefringence is mainly attributed to the intermolecular structure dynamics of water.

### Theoretical model

The measured phase shift Δ*φ* is positively correlated with the birefringence Δ*n*, which can be divided into the electronic contribution and molecular contribution. The electronic contribution can be expressed as the multiplication of the square of the electric field with the wavelength and response factor *B*_*e*_^32,37^, i.e., $\Delta n_e = \lambda B_e\left| {E(z,t)} \right|^2$. For the molecular contribution, both the relaxation process and hydrogen bond vibration process contribute to the birefringence signal, i.e., $\Delta n_m = \Delta n_D + \Delta n_i$ (*i* = 1 and *i* = 2 represent the hydrogen bond bending and stretching vibrations, respectively). Δ*n*_*D*_ is the birefringence caused by the Debye relaxation process, and its intrinsic molecular dynamics have been well explained in previous studies^32,38,39^. However, no study has systematically investigated the contribution to Δ*n*_*i*_ that is related to intermolecular hydrogen bond motions.

**Fig. 2:**
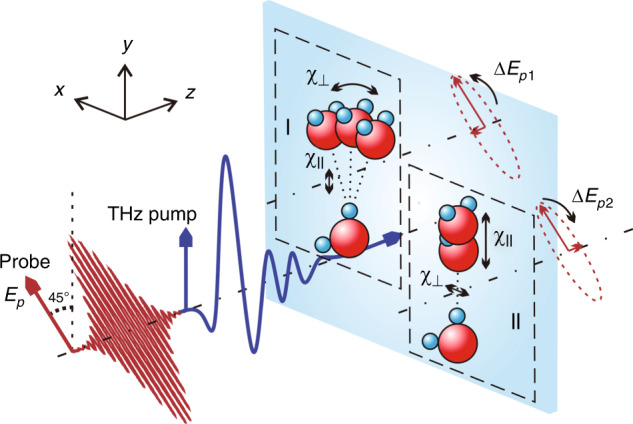
The effect of hydrogen bond vibrations on the dielectric susceptibility anisotropy. The direction of the hydrogen bond is defined as the parallel direction. (I) In this hydrogen bond unit, if only the bending mode is considered, then the dielectric susceptibility anisotropy is negative (*χ*_||_ < *χ*_⊥_) because the dielectric susceptibility in the vertical direction (*χ*_⊥_) is disturbed. (II) In contrast, if only the stretching mode is considered, then the dielectric susceptibility in the parallel direction (*χ*_||_) is disturbed. As a result, a linearly polarized probe pulse (*E*_*p*_) traversing liquid water could acquire ellipticity with opposite handedness because of the two types of molecular motions. A bipolar TKE response combining these two influences is expected

Here, the Lorentz dynamic equation is adopted in our model, which is based on the perturbation in the dielectric tensor caused by the intermolecular vibration modes. A spatial rectangular coordinate system is established, as shown in Fig. 2. The beam propagation direction is set as the *z*-axis, and the THz polarization direction is set as the *y*-axis. We use *Q*_*b*_ and *Q*_*s*_ to represent the bending and stretching vibration amplitudes of a single hydrogen bond unit, respectively. *Q*_1_ = 〈*Q*_*b*_〉 and *Q*_2_ = 〈*Q*_*s*_〉 represent the average amplitude tensors of the hydrogen bond bending and stretching modes per unit volume, respectively. Without the THz pump electric field, the water molecular motions are in an isotropic thermodynamic equilibrium state, and the amplitudes of the hydrogen bond vibration in all directions are equal. When a THz electric field is applied in the y direction, the instantaneous rotation of water molecules provides an additional driving potential for the intermolecular hydrogen bond bending and stretching vibrations^14,32^.

According to the Taylor series, in one hydrogen bond unit, the perturbations of the dielectric susceptibilities parallel and perpendicular to the hydrogen bond direction caused by the intermolecular modes are expressed as follows (see the “Materials and methods” section for details):

1 $$\chi _\parallel = \chi _0 + \frac{{\partial \chi _\parallel }}{{\partial Q_b}}Q_b + \frac{{\partial \chi _\parallel }}{{\partial Q_s}}Q_s,\,\,\chi _ \bot = \chi _0 + \frac{{\partial \chi _ \bot }}{{\partial Q_b}}Q_b + \frac{{\partial \chi _ \bot }}{{\partial Q_s}}Q_s$$

For the bending mode, the hydrogen bond oscillator mainly perturbs the dielectric susceptibility component vertical to the hydrogen bond direction, which is represented by phenomenon (I) in Fig. 2, i.e., $\frac{{\partial \chi _\parallel }}{{\partial Q_b}}Q_b \ll \frac{{\partial \chi _ \bot }}{{\partial Q_b}}Q_b$, where $\frac{{\partial \chi _\parallel }}{{\partial Q_b}}Q_b$ can be ignored. Similarly, the perturbation of the dielectric susceptibility caused by the stretching vibration is mainly in the parallel direction with respect to the hydrogen bond, which is depicted by phenomenon (II) in Fig. 2. Therefore, the perturbation of the dielectric susceptibility vertical to the hydrogen bond direction mainly originates from the bending vibration, i.e., $\chi _ \bot \approx \chi _0 + \frac{{\partial \chi _ \bot }}{{\partial Q_b}}Q_b$, and that parallel to the hydrogen bond direction mainly comes from the stretching vibration, i.e., $\chi _\parallel \approx \chi _0 + \frac{{\partial \chi _\parallel }}{{\partial Q_s}}Q_s$.

The macroscopic expression of the dielectric tensor is the average value of all the vectors of the microscopic units per unit volume. The driving THz electric field (y-polarized) will cause instantaneous rotation of the water molecule and provide a driving potential in the y direction. Due to the directional relationship between the hydrogen bond and the permanent dipole moment of the water molecule, the y component of the hydrogen bond will obtain more coupling energy^24^. The anisotropic perturbation caused by the intermolecular vibration modes excited by the y-polarized THz electric field is defined as $q_i = Q_iy - \frac{1}{2}(Q_ix + Q_iz) > 0$ (*q*_1_ and *q*_2_ represent the anisotropic perturbations caused by hydrogen bond bending and stretching vibrations, respectively). The resulting polarizability anisotropy can be expressed by the refractive index associated with the dielectric susceptibility *χ* (see the “Materials and methods” section for details)^40^:

2 $$\mathop {\sum}\limits_{i = 1,2} {\Delta n_i} = \frac{1}{{2\varepsilon _0n_0}}\left[ {\varepsilon _y - \frac{1}{2}(\varepsilon _x + \varepsilon _z)} \right] = \frac{1}{{2n_0}}\left( {\frac{{\partial \chi _\parallel }}{{\partial Q_s}}q_2 - \frac{{\partial \chi _ \bot }}{{\partial Q_b}}q_1} \right)$$

where *ε*_0_ is the vacuum dielectric constant and *n*_0_ is the refractive index of liquid water. *q*_*i*_ mentioned above is positive. These two intermolecular motions result in opposite birefringence contributions to the total refractive index, i.e., $\Delta n_1 < 0,\,\Delta n_2 > 0$.

In addition, *q*_*i*_ satisfies the Lorentz dynamic model, which describes the motion of the damped harmonic oscillator^41,42^ (see the “Materials and methods” section for details):

3 $$\frac{{\partial ^2q_i(t)}}{{\partial t^2}} + \gamma _i\frac{{\partial q_i(t)}}{{\partial t}} + \omega _i^2q_i(t) = a_iE^2(t)\quad (i = {\mathrm{1,2}})$$

where *γ*_*i*_, *ω*_*i*_ represent the damping coefficient and inherent frequency, respectively, with values^15^ of *γ*_1_ = 115 cm^−1^, *γ*_2_ = 165 cm^−1^, *ω*_1_ = 60 cm^−1^ and *ω*_2_ = 190 cm^−1^. Here, $a_i = \beta _i\frac{{\mu _0^2}}{{3\sqrt 2 k_BTm}}$ represents the coupling factor between the square of the THz pump electric field and the driving term in Eq. (3). A detailed description of *a*_*i*_ can be found in the “Materials and methods” section.

### Simulation and analysis

**Fig. 3:**
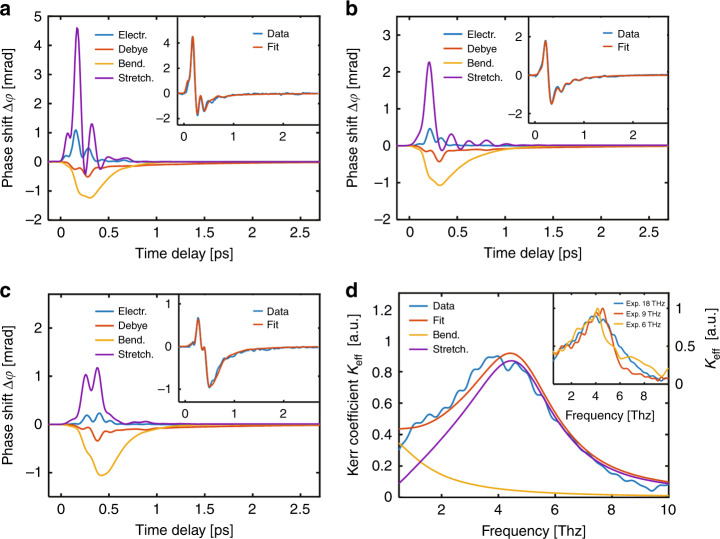
Simulation results of measurement data. Theoretical simulation of the TKE response in the time domain for the electronic, Debye relaxation, hydrogen bond stretching vibration and hydrogen bond bending vibration contributions under different pump electric fields with cut-off frequencies of **a** 18THz, **b** 9THz and **c** 6THz. The corresponding inset shows a comparison between the sum of all the contributions and the measured data. **d** Theoretical frequency-domain Kerr coefficient *K*_eff_ under hydrogen bond bending (yellow line) and stretching (purple line) modes. The sum of these two theoretical Kerr coefficients (red line) closely matches the data extracted from the experimental results with a cut-off frequency of 18THz (blue line). The inset shows the measured Kerr coefficient *K*_eff_ extracted from the birefringent signals under different electric fields with cut-off frequencies of 18, 9 and 6THz

We use the above model to decompose and simulate the experimental data in the time domain, as shown in Fig. 3a–c. The data can also be decomposed into (i) positive responses with electronic (blue line) and hydrogen bond stretching (purple line) contributions and (ii) negative responses with Debye relaxation (red line) and hydrogen bond bending (yellow line) contributions. To validate our proposed model, we change the frequency range of the THz pump pulse by using different low-pass filters (LPFs). As shown in Fig. 3a–c, the simulation results based on our proposed model are in good agreement with the measured data. As the cut-off frequency decreases, the positive polarity response is more significantly reduced than the negative polarity response, indicating that the positive response is mainly derived from higher-frequency molecular motion. In addition, the weak electronic contribution of water can be attributed to the very small hyperpolarizabilities in water^32,43^.

We find that the molecular contribution exhibits a strong frequency dependence due to the participation of pertinent molecular motion mechanisms. Here, we use an ultrabroadband THz pulse to excite liquid water, and the induced transient anisotropy is dominated by the intermolecular hydrogen bond stretching and bending vibrations. As the frequency decreases, the ratio of the anisotropic contribution caused by the intermolecular modes to the electronic contribution increases. This phenomenon is reasonable for intermolecular modes with characteristic frequencies less than 6 THz. Moreover, the latest experiment on THz-driven liquid water shows that with a lower centre frequency excitation (~0.25 THz), the orientation process guides the anisotropic distribution, showing a negative TKE response^32^. However, Kampfrath et al. observed a bipolar Kerr effect of liquid water with an ~1 THz centre frequency for excitation^31^, which suggested that the molecular response mechanism depends critically on the driving frequency. For a relatively high THz frequency excitation (such as ~3.9 THz centre frequency), the molecular orientation process loses its dominant position, and intermolecular modes with obvious damping characteristics dominate. It can be predicted that the librational modes need to be considered when using a pulse with a higher THz frequency excitation (such as frequencies above 12 THz)^16^.

We mainly attribute the bipolar TKE signal to the contributions of the two distinct modes of hydrogen bond vibrations rather than to the electronic or Debye contribution. Here, we perform a Fourier transform of the homogeneous solutions of the Lorentz time-domain differential equations for hydrogen bond bending and stretching modes in our model and obtain the theoretical frequency-domain Kerr coefficient in these two intermolecular modes, as shown by the yellow and purple lines in Fig. 3d. Moreover, we extract the frequency-domain Kerr coefficient *K*_eff_ (${\it{{\cal{F}}}}(\Delta n(t)){\mathrm{/}}{\it{{\cal{F}}}}(E^2(t))$) from our experimental results for the three input THz electric fields over the range of 0.5–10 THz, and this coefficient has a high signal-to-noise ratio and mainly covers the intermolecular modes. A significant peak appears near 4.5 THz and corresponds to the contribution of the hydrogen bond stretching vibration. Since the hydrogen bond bending vibration is approximately a critically damped harmonic oscillation, no apparent peak position is observed. Similarly, for the TKE time-domain responses of liquid water, the apparent oscillation features are primarily due to the underdamped harmonic oscillation of stretching. We find that the measured Kerr coefficient (blue line) can be approximated by the sum of the two intermolecular mode contributions (red line). The Kerr coefficient response induced by intermolecular hydrogen bond vibrations can be simply approximated by the following formula:

4 $$K_{{\mathrm{eff}}}(\omega ) = \mathop {\sum}\limits_{i = 1,2} {\frac{{\left| {k_i} \right|}}{{\omega _i^2 - \omega ^2 + i\gamma _i\omega }}}$$

where the real part of *K*_eff_ is the Kerr coefficient. *k*_*i*_ is the scaling constant of the THz Kerr effect^44^, i.e., $k_1 = \frac{{a_1}}{\lambda }\left( {\frac{{\partial \chi _\parallel }}{{\partial Q_b}} - \frac{{\partial \chi _ \bot }}{{\partial Q_b}}} \right)$ and $k_2 = \frac{{a_2}}{\lambda }\left( {\frac{{\partial \chi _\parallel }}{{\partial Q_s}} - \frac{{\partial \chi _ \bot }}{{\partial Q_s}}} \right)$. *k*_1_ and *k*_2_ calculated from the experimental data are $k_1 \approx - 7 \times {\mathrm{10}}^9\,{\mathrm{C/}}({\mathrm{V}} \cdot {\mathrm{kg}} \cdot {\mathrm{m}})$ and $k_2 \approx 6.5 \times {\mathrm{10}}^{10}\,{\mathrm{C/}}({\mathrm{V}} \cdot {\mathrm{kg}} \cdot {\mathrm{m}})$, respectively.

**Fig. 4:**
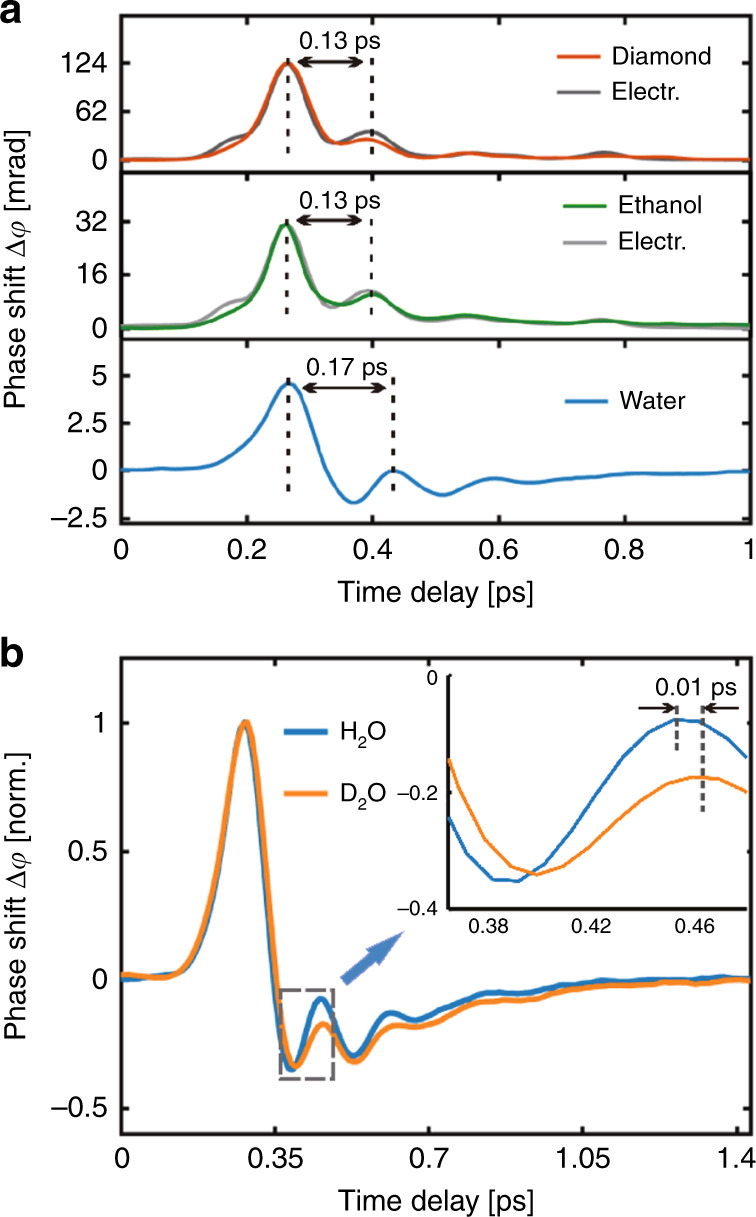
TKE responses of different materials. **a** The TKE responses of diamond (top-red) and ethanol (middle-green) exhibit similar double-peak structures with a time interval of 0.13ps. The simulated electronic responses of diamond and ethanol are shown as dark grey and light grey lines, respectively. In comparison, the TKE response of liquid water (bottom) comprises a broadened double-peak structure with a time interval of 0.17ps and an obvious negative component. **b** The TKE responses of liquid water and heavy water are demonstrated for comparison

To further confirm that our birefringent signal of liquid water mainly comes from unique hydrogen bond motions rather than the electronic contribution, we carry out comparative measurements of ethanol, diamond and water under a pump electric field with a cut-off frequency of 18 THz. The TKE signals of diamond and ethanol exhibit a double-peak structure, which is a typical Kerr-type response curve, as shown in the top and middle panels of Fig. 4a. In comparison, the TKE signal of liquid water has not only a distinct negative component but also a broadened double-peak structure, as shown in the bottom panel of Fig. 4a. Theoretically, diamond as an ultrafast pulse switching, its TKE response curve will fully follow the square of the THz field^45,46^. Here, we simulate the electronic response by considering the influence factors of sampling error and the THz wave transmission process in the sample (such as the phase shift of a THz wave during its transmission in the sample, the attenuation of THz intensity in the sample, and the dispersion between the THz wave and the probe light in the sample)^35,46,47^. We obtain the expected fitting curve of the diamond’s electronic response, which is in good agreement with the experimental data. Similarly, the simulated electronic response of ethanol agrees well with the experimental data, revealing that the TKE signal of ethanol is mainly dominated by the electronic response. It is worth noting that Kampfrath et al. also observed that the TKE signal of ethanol is dominated by the electronic response under a pump THz field with ~1 THz centre frequency^27^. However, Zalden et al. used THz pulses with ~0.25 THz centre frequency to excite ethanol and obtained a relatively high molecular response^32^, revealing that the molecular response of ethanol depends heavily on the driving frequency^48^. Compared with diamond and ethanol, water has a much weaker electronic response (as shown in Fig. 3a), which is due to the small effective medium length (~25-µm thick water can absorb 90% of THz energy in our experiment) and the small electronic response factor *B*_*e*_ (*B*_*e*_: <0.003 × 10^−14^ m/V^2^ for liquid water, ~0.00996 × 10^−14^ m/V^2^ for diamond, and ~0.0093 × 10^−14^ m/V^2^ for ethanol)^32,43,46^. In addition, the simulated electronic response of water is closer to the shape of the THz intensity curve, mainly because the small effective medium length limits the distortion of the electronic response introduced by the THz wave transmission process in the sample. Moreover, the THz intensity curve with ~3.9 THz centre frequency has the characteristic of a double-peak time interval of 0.13 ps, and this characteristic is also displayed in the electronic-type TKE response curves of diamond and ethanol. In comparison, the TKE signal of water exhibits a broadened double-peak time interval of 0.17 ps, which represents the damped oscillation period corresponding to the hydrogen bond stretching vibration with a characteristic frequency of 5.7 THz. In summary, we believe that the unique TKE response of water with the broadened double-peak characteristic mainly originates from the contribution of intermolecular modes rather than the electronic response.

To verify the applicability of our model, we measure the TKE response of heavy water and show it in Fig. 4b. Compared with that of water, the characteristic frequency of the hydrogen bond stretching vibration of heavy water exhibits a slight red shift^15^, which results in a slight broadening of the double-peak time interval of the TKE response. In addition, the relatively large damping coefficient of heavy water in the stretching mode corresponds to the faster energy decay process of the harmonic oscillator, resulting in a reduction in the second peak of the TKE response compared to that of water. Moreover, our model is used in frequency-dependent simulations of heavy water and matches well with experimental results (see section SIII in the Supplementary Information for details).

## Discussion

We use a THz electric field to resonantly excite the intermolecular modes of liquid water. We believe that the transient rotation of a molecule produces an induced dipole moment, which immediately transfers the momentum driven by the THz field to the restricted translational motion of adjacent water molecules. This translational motion can be assigned to a bending mode and a stretching mode, which can lead to the components of polarizability anisotropy perpendicular and parallel to the hydrogen bonds, respectively, thus resulting in bidirectional performance. This assignment is also confirmed by Raman spectroscopy^49^.

In recent studies of THz-excited reorientational diffusive motions in liquid water, the authors explained the negative contribution origins from molecular orientation or intermolecular motion due to a low-frequency THz pulse (<1 THz centre frequency)^31,32^. However, they described the measured positive component simply as a fast relaxation process with exponential decay^31^. In our experiment, the measured TKE response shows a distinctly damped oscillation process with a 170 fs double-peak time interval, indicating that this phenomenon cannot be explained by a fast relaxation process. Moreover, the sub-picosecond time scale of 170 fs is consistent with the characteristic frequency of the hydrogen bond stretching vibration, revealing that the positive Kerr effect is mainly derived from the stretching vibration rather than a fast relaxation process. Furthermore, compared with the negative response with a relaxation tail extending over a few picoseconds reported in the literature^31,32^, our negative response exhibits a feature on the sub-picosecond scale. Through our theoretical simulation, we conclude that this feature is mainly derived from a damped oscillation process of the bending mode rather than a molecular orientation process.

We infer that our method has the potential to detect the physical mechanism of damped harmonic oscillator processes at higher frequencies^16^, such as 420 and 620 cm^−1^. However, due to the response function of our instrument, consideration of frequencies higher than 400 cm^−1^ is not necessary. In addition, according to the multicomponent schematic formulation of the main mode-coupling theory (SMC)^22^, high-frequency vibrational contributions (>400 cm^−1^) do not affect the response function in the lower-frequency range that our work investigated.

In conclusion, we have demonstrated that the low-frequency molecular motion patterns of liquid water molecules are excited by an intense and ultrabroadband THz electric field. The TKE response is assigned to the superposition of four components, among which the bidirectional contributions of bending and stretching modes play dominant roles. We proposed a hydrogen bond oscillator model based on the Lorentz dynamic equation to describe the dynamics of the intermolecular modes of liquid water and successfully reproduced the measured TKE responses. A Kerr coefficient response equation was further established to study the influence of the underlying parameters. We believe that our method can also be applied to detect the physical mechanisms of the gas phase of water and crystalline and amorphous ices, as well as the complex interaction of reagents with solvent water molecules.

## Materials and methods

### Experimental setup

The experimental setup is schematically depicted in Fig. S1 in the Supplementary Information. The laser produced by a 5 mJ Ti:sapphire laser system (Spitfire, Spectra Physics) with a central wavelength of 800 nm and a repetition rate of 1 kHz is separated into two beams by a beam splitter with a 7:3 (R:T) split ratio. One of the 3.5 mJ beams is used to pump a commercial optical parametric amplifier (TOPAS, Spectra Physics), which delivers an optical laser beam with a wavelength of 1550 nm. The other beam is used as a probe laser with a wavelength of 800 nm. An organic 4-N,N-dimethylamino-4′-N′-methyl-stilbazoliumtosylate (DAST) crystal (Swiss Terahertz LLC) is pumped by the optical beam with a 1550 nm wavelength, and a THz wave is collinearly generated. Residual NIR is filtered out using three low-pass filters (QMC Instruments Ltd) with cut-off frequencies of 18 THz. The THz beam is expanded and then focused by three off-axis parabolic mirrors. The energy of the THz wave is controlled by two THz polarizers (POL-HDPE-CA50-OD63-T8, Tydex). Two aluminium wires with diameters of 100 µm are separated by ~5 mm to form a stable free-flowing water film. The thickness of the water film is 90 ± 4 µm and can be adjusted by controlling the water flow rate. Our stable free-flowing water film can effectively eliminate the heat accumulation between two adjacent THz pump pulses, and the contribution of heating effects to the TKE signal in the experiment is negligible (see section SVI in the Supplementary Information for details). An optical second-harmonic intensity autocorrelator is used to measure and calibrate the thickness^50^.

The linearly polarized THz pulse is focused on liquid water. The polarization of the probe beam with an 800 nm wavelength is tilted by 45° relative to the THz electric field polarization. The time trajectory is recorded by scanning the delay (Here, an electric stage type M-ILS100PP purchased from Newport Corporation is used.) between the pump and probe beams and reading the modulation caused by the THz pump pulse detected by a balanced photodiode (PDB210A/M, Thorlabs) using a lock-in amplifier (Model SR830 DSP, Stanford Research Systems). The system is purged with dry nitrogen gas to eliminate the absorption of water vapour. However, nitrogen gas has a small TKE response under our experimental conditions. Therefore, the background response of nitrogen gas has been subtracted from the data presented in the article (see section SV in the Supplementary Information for details). The resulting birefringence Δ*n*(*z*, *t*) is detected based on the phase shift Δ*φ*(*t*) at position *z* along the propagation direction^51,52^ via:

5 $$\Delta \varphi (t) = \frac{{2\pi }}{\lambda }{\int_0^l} {\Delta n(z,t)dz}$$

### Sample source

Ultra-pure water (*σ* < 0.1 μS/cm) is obtained from a lab-based purification system. Heavy water with 99% purity is commercially obtained from CIL (USA). Ethanol with >99.8% purity is commercially obtained from HUSHI (CHN). In our work, both water and ethanol have a thickness of 90 µm by using a stable free-flowing liquid film device. The diamond substrate is a single crystal and has a thickness of 0.3 mm. It is purchased from Element Six (UK). During all measurements, the surrounding environment is held at a constant temperature of 22 ± 1 °C.

### Model details

The perturbation of the dielectric susceptibility caused by molecular vibrations can be expanded into the following Taylor series according to the components of the amplitude:

6 $$\chi = \chi _0 + \mathop {\sum}\limits_i {\frac{{\partial \chi }}{{\partial Q_i}}Q_i + \frac{1}{2}\mathop {\sum}\limits_{i,j} {\frac{{\partial ^2\chi }}{{\partial Q_1\partial Q_2}}} Q_iQ_j} + ......$$

Then, the dielectric function can be written as *ε* = *ε*_0_(*ε*_*r*_ + *χ*), where *ε*_*r*_ is the relative dielectric constant of the 800 nm probe pulse without THz wave pumping. Through experimental data, we find that the signal response has good linearity with the square of the electric field, which proves that the polarization anisotropy of water molecules is mainly derived from second-order polarization and has no higher-order terms. Therefore, the above formula can be simplified as:

7 $$\chi = \chi _0 + \mathop {\sum}\limits_i {\frac{{\partial \chi }}{{\partial Q_i}}Q_i}$$

In this work, the intermolecular hydrogen bond motions of liquid water have two significant oscillator vibration modes in the THz range: bending vibration and stretching vibration. The dielectric tensor after the spindle transformation is *ε* = [*ε*_*x*_*ε*_*y*_*ε*_*z*_]^*T*^. According to Eq. (7), the dielectric tensor can be expressed as:

8 $$\left[ \begin{array}{l}\varepsilon _x\\ \\ \varepsilon _y\\ \\ \varepsilon _z\end{array} \right] = \varepsilon _0[1 + \chi _0]I + \varepsilon _0\left\{ {\left[ \begin{array}{l}\frac{{\partial \chi _\parallel }}{{\partial Q_b}},{\mathrm{ }}\frac{{\partial \chi _ \bot }}{{\partial Q_b}},{\mathrm{ }}\frac{{\partial \chi _ \bot }}{{\partial Q_b}}\\ \frac{{\partial \chi _ \bot }}{{\partial Q_b}},{\mathrm{ }}\frac{{\partial \chi _\parallel }}{{\partial Q_b}},{\mathrm{ }}\frac{{\partial \chi _ \bot }}{{\partial Q_b}}\\ \frac{{\partial \chi _ \bot }}{{\partial Q_b}},{\mathrm{ }}\frac{{\partial \chi _ \bot }}{{\partial Q_b}},{\mathrm{ }}\frac{{\partial \chi _\parallel }}{{\partial Q_b}}\end{array} \right]\left[ \begin{array}{l}Q_1x\\ \\ Q_1y\\ \\ Q_1z\end{array} \right] + \left[ \begin{array}{l}\frac{{\partial \chi _\parallel }}{{\partial Q_s}},{\mathrm{ }}\frac{{\partial \chi _ \bot }}{{\partial Q_s}},{\mathrm{ }}\frac{{\partial \chi _ \bot }}{{\partial Q_s}}\\ \frac{{\partial \chi _ \bot }}{{\partial Q_s}},{\mathrm{ }}\frac{{\partial \chi _\parallel }}{{\partial Q_s}},{\mathrm{ }}\frac{{\partial \chi _ \bot }}{{\partial Q_s}}\\ \frac{{\partial \chi _ \bot }}{{\partial Q_s}},{\mathrm{ }}\frac{{\partial \chi _ \bot }}{{\partial Q_s}},{\mathrm{ }}\frac{{\partial \chi _\parallel }}{{\partial Q_s}}\end{array} \right]\left[ \begin{array}{l}Q_2x\\ \\ Q_2y\\ \\ Q_2z\end{array} \right]} \right\}$$

Therefore, through the deduction of the above formula, the polarization anisotropy Δ*n*_*i*_ caused by THz wave driving can be expressed as Eq. (2).

For an oscillator with amplitude *Q*, mass *m*, and inherent frequency *ω*, the molecular harmonic oscillator equation can be expressed as^41,42^:

9 $$\frac{{\partial ^2Q}}{{\partial t^2}} + \gamma \frac{{\partial Q}}{{\partial t}} + \omega ^2Q = \frac{{\partial W}}{{m\partial Q}}$$

where *W* is the potential energy of the oscillator driven by the electric field. In this model, the potential energy of the THz electric field for driving the instantaneous rotation of water molecules is *E* · *μ*_ind_. The induced dipole moment caused by the instantaneous rotation of the permanent dipole moment of a water molecule can be expressed as $\mu _{{\mathrm{ind}}} = \Delta \alpha E = \mu _0\left\langle {\cos \theta } \right\rangle$, where Δ*α* = *α*_||_ − *α*_⊥_ means that the polarizability of the water molecule parallel to the direction of the permanent dipole moment is smaller than the polarizability perpendicular to the permanent dipole moment. We can establish the relationship between $\left\langle {\cos \theta } \right\rangle$ and the THz electric field *E* based on the theory of Kalmykov and Coffey^38,39^:

10 $$\left\langle {\cos \theta } \right\rangle \approx \frac{{\left| {\mu _0} \right|}}{{3\sqrt 2 k_BT}}E(t) \ast R(t)$$

Due to the instantaneous rotation of water molecules during synergy with the hydrogen bond oscillator, the coupling potential energy obtained by the hydrogen bond oscillator is positively correlated with its amplitude. Therefore, the potential energy can be given as:

11 $$W(t) \approx \beta Q\frac{{\mu _0^2}}{{3\sqrt 2 k_BT}}R(t) \ast E^2(t)$$

where *β* (m^−1^) is the coupling coefficient and *R*(*t*) represents the fast Debye relaxation process of water molecules, which has a time constant of ~0.1 ps; thus, the right-hand side of the harmonic oscillator equation can be simply given as $\frac{{\partial W}}{{m\partial Q}} \approx \beta \frac{{\mu _0^2}}{{3\sqrt 2 k_BTm}}E^2(t)$. Since *q*_*i*_ is linearly related to *Q*, *q*_*i*_ also satisfies the Lorentz oscillation process described by Eq. (3), and in this case, the driving term on the right-hand side of Eq. (3) can be given as *a*_*i*_*E*^2^(*t*), where $a_i = \beta _i\frac{{\mu _0^2}}{{3\sqrt 2 k_BTm}}$.

## Supplementary information

Supplementary Information

## References

[1] Cisneros GA (2016). Modeling molecular interactions in water: from pairwise to many-body potential energy functions. Chem. Rev..

[2] Bellissent-Funel MC (2016). Water determines the structure and dynamics of proteins. Chem. Rev..

[3] Omta AW (2003). Negligible effect of ions on the hydrogen-bond structure in liquid water. Science.

[4] Fecko CJ (2003). Ultrafast hydrogen-bond dynamics in the infrared spectroscopy of water. Science.

[5] Smith JD (2004). Energetics of hydrogen bond network rearrangements in liquid water. Science.

[6] Stiopkin IV (2011). Hydrogen bonding at the water surface revealed by isotopic dilution spectroscopy. Nature.

[7] Richardson JO (2016). Concerted hydrogen-bond breaking by quantum tunneling in the water hexamer prism. Science.

[8] Stokely K (2010). Effect of hydrogen bond cooperativity on the behavior of water. Proc. Natl Acad. Sci. USA.

[9] Sharma M, Resta R, Car R (2005). Intermolecular dynamical charge fluctuations in water: a signature of the H-bond network. Phys. Rev. Lett..

[10] Heyden M, Tobias DJ (2013). Spatial dependence of protein-water collective hydrogen-bond dynamics. Phys. Rev. Lett..

[11] Bakker HJ, Skinner JL (2010). Vibrational spectroscopy as a probe of structure and dynamics in liquid water. Chem. Rev..

[12] Perakis F (2016). Vibrational spectroscopy and dynamics of water. Chem. Rev..

[13] Vij JK, Simpson DRJ, Panarina OE (2004). Far infrared spectroscopy of water at different temperatures: GHz to THz dielectric spectroscopy of water. J. Mol. Liq..

[14] Torii H (2011). Intermolecular electron density modulations in water and their effects on the far-infrared spectral profiles at 6 THz. J. Phys. Chem. B.

[15] Mizoguchi K, Hori Y, Tominaga Y (1992). Study on dynamical structure in water and heavy water by low-frequency Raman spectroscopy. J. Chem. Phys..

[16] Fukasawa T (2005). Relation between dielectric and low-frequency Raman spectra of hydrogen-bond liquids. Phys. Rev. Lett..

[17] Rønne C, Keiding SR (2002). Low frequency spectroscopy of liquid water using THz-time domain spectroscopy. J. Mol. Liq..

[18] Penkov N (2015). Terahertz spectroscopy applied for investigation of water structure. J. Phys. Chem. B.

[19] Savolainen J, Ahmed S, Hamm P (2013). Two-dimensional Raman-terahertz spectroscopy of water. Proc. Natl Acad. Sci. USA.

[20] Torre R, Bartolini P, Righini R (2004). Structural relaxation in supercooled water by time-resolved spectroscopy. Nature.

[21] Turton DA, Wynne K (2008). Structural relaxation in the hydrogen-bonding liquids N-methylacetamide and water studied by optical Kerr effect spectroscopy. J. Chem. Phys..

[22] Taschin A (2013). Evidence of two distinct local structures of water from ambient to supercooled conditions. Nat. Commun..

[23] Winkler K, Lindner J, Vöhringer P (2002). Low-frequency depolarized Raman-spectral density of liquid water from femtosecond optical Kerr-effect measurements: lineshape analysis of restricted translational modes. Phys. Chem. Chem. Phys..

[24] Sajadi M, Wolf M, Kampfrath T (2017). Transient birefringence of liquids induced by terahertz electric-field torque on permanent molecular dipoles. Nat. Commun..

[25] Freysz E, Degert J (2010). Nonlinear optics: terahertz Kerr effect. Nat. Photon..

[26] Finneran IA (2016). Coherent two-dimensional terahertz-terahertz-Raman spectroscopy. Proc. Natl Acad. Sci. USA.

[27] Kampfrath T (2018). The nature of the dielectric response of methanol revealed by the terahertz Kerr effect. J. Phys. Chem. Lett..

[28] Hoffmann MC (2009). Terahertz kerr effect. Appl. Phys. Lett..

[29] Bodrov S (2017). Terahertz induced optical birefringence in polar and nonpolar liquids. J. Chem. Phys..

[30] Allodi MA, Finneran IA, Blake GA (2015). Nonlinear terahertz coherent excitation of vibrational modes of liquids. J. Chem. Phys..

[31] Kampfrath, T., Wolf, M. & Sajadi, M. Anharmonic coupling between intermolecular motions of water revealed by terahertz Kerr effect. Preprint at https://arxiv.org/abs/1707.07622 (2017).

[32] Zalden P (2018). Molecular polarizability anisotropy of liquid water revealed by terahertz-induced transient orientation. Nat. Commun..

[33] Novelli, F. et al. Strong anisotropy in liquid water upon librational excitation using terahertz laser fields. Preprint at https://arxiv.org/abs/1809.04261 (2018). 10.1021/acs.jpcb.0c02448 32450043

[34] Zasetsky AY (2011). Dielectric relaxation in liquid water: two fractions or two dynamics?. Phys. Rev. Lett..

[35] Hale GM, Querry MR (1973). Optical constants of water in the 200-nm to 200-µm wavelength region. Appl. Opt..

[36] Afsar MN, Hasted JB (1977). Measurements of the optical constants of liquid H_2_O and D_2_O between 6 and 450 cm^−1^. J. Opt. Soc. Am..

[37] Maroulis G (1998). Hyperpolarizability of H_2_O revisited: accurate estimate of the basis set limit and the size of electron correlation effects. Chem. Phys. Lett..

[38] Kalmykov YP (2009). Matrix method calculation of the Kerr effect transient and ac stationary responses of arbitrary shaped macromolecules. J. Chem. Phys..

[39] Coffey WT, Kalmykov YP (2012). The Langevin Equation.

[40] Boyd RW (2003). Nonlinear Optics.

[41] Demtröder, W. *Laser Spectroscopy: Vol. 2 Experimental Techniques.* (Springer, Berlin, Heidelberg, 2008).

[42] Levenson M (2012). Introduction to Nonlinear Laser Spectroscopy..

[43] Sekino H, Bartlett RJ (1993). Molecular hyperpolarizabilities. J. Chem. Phys..

[44] Palese S (1994). Femtosecond optical Kerr effect studies of water. J. Phys. Chem..

[45] Shalaby M, Vicario C, Hauri CP (2017). Extreme nonlinear terahertz electro-optics in diamond for ultrafast pulse switching. APL Photon..

[46] Sajadi M, Wolf M, Kampfrath T (2015). Terahertz-field-induced optical birefringence in common window and substrate materials. Opt. Express.

[47] Ahmed S, Savolainen J, Hamm P (2014). The effect of the Gouy phase in optical-pump-THz-probe spectroscopy. Opt. Express.

[48] Amo Y, Tominaga Y (2000). Low-frequency Raman study of ethanol–water mixture. Chem. Phys. Lett..

[49] Fecko CJ, Eaves JD, Tokmakoff A (2002). Isotropic and anisotropic Raman scattering from molecular liquids measured by spatially masked optical Kerr effect spectroscopy. J. Chem. Phys..

[50] Jin Q (2017). Observation of broadband terahertz wave generation from liquid water. Appl. Phys. Lett..

[51] Gallot G, Grischkowsky D (1999). Electro-optic detection of terahertz radiation. J. Opt. Soc. Am. B.

[52] Leitenstorfer A (1999). Detectors and sources for ultrabroadband electro-optic sampling: experiment and theory. Appl. Phys. Lett..

