# Supplementary material for: Ultrafast hydrogen bond dynamics of liquid water revealed by terahertz-induced transient birefringence
Source: Light Sci Appl. 2020 Aug 4;9:136. doi: 10.1038/s41377-020-00370-z (PMC7403349; doi:10.1038/s41377-020-00370-z)
Supplement: Supplementary file 1 — Supplementary Information [file 41377_2020_370_MOESM1_ESM.pdf]

Supplementary Information for

Ultrafast Hydrogen Bond Dynamics of Liquid Water Revealed by Terahertz-induced Transient Birefringence

Authors
Hang Zhao[1,+], Yong Tan[1,+], Liangliang Zhang[2,*], Rui Zhang[3], Mostafa Shalaby[2], Cunlin Zhang[2], Yuejin Zhao[1,*], and Xi-Cheng Zhang[4]

Affiliations
[1]Beijing Key Laboratory for Precision Optoelectronic Measurement Instrument and Technology, School of Optics and Photonics, Beijing Institute of Technology, Beijing 100081, China
[2]Beijing Advanced Innovation Center for Imaging Technology and Key Laboratory of Terahertz Optoelectronics (MoE), Department of Physics, Capital Normal University, Beijing 100048, China
[3]Shenzhen Institutes of Advanced Technology, Chinese Academy of Sciences, Shenzhen, 518055, China
[4]The Institute of Optics, University of Rochester, Rochester, NY 14627, USA
*Correspondence: Liangliang Zhang (zhlliang@126.com) or Yuejin Zhao (yjzhao@bit.edu.cn)
+These authors contributed equally to this paper.

Supplementary information

SI. Experimental setup and correction of the THz signal detected from GaP

The experimental setup is schematically depicted in Fig. S1. Regarding the acquisition of the THz electric field waveform, we use electro-optical sampling with 100 μm GaP. Because the intense THz pulse saturates the GaP response, we use the THz Kerr signal of diamond to calibrate the THz electric field strength[1-3]. For an ultrabroadband THz pulse, it is additionally distorted by frequency-dependent phase matching, reflection, dispersive propagation, and absorption in the electro-optic detection crystal. Therefore, we use the full complex response function of GaP detector to reconstruct all the spectra presented in this work[4-7], and the obtained data are shown in Fig. S2. As a result, we obtain an intense (with peak electric field E=14.9 MV cm^{-1}) and ultrabroadband (over the range of 1-10 THz with a center frequency of 3.9 THz) THz pulse.

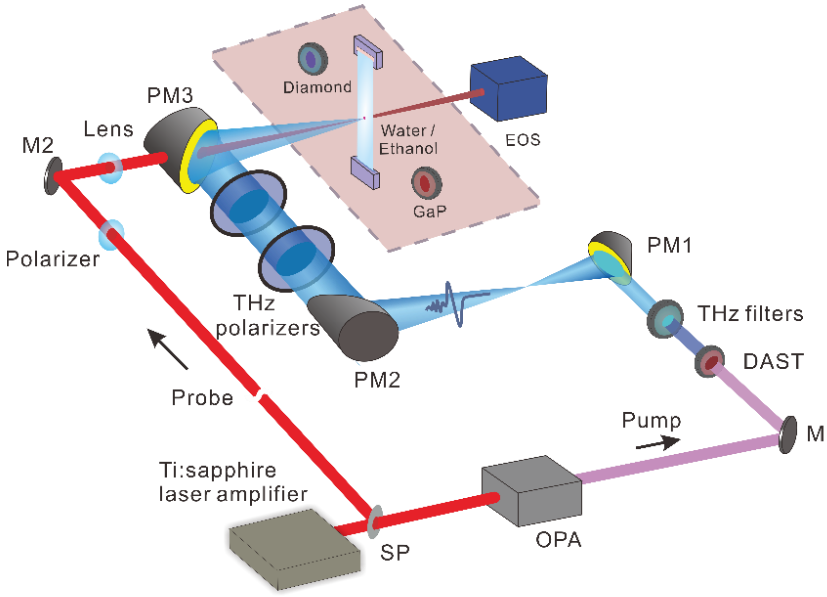

Fig. S1 Diagram of the experimental system (SP: splitter; OPA: optical parametric amplifier; M: mirror; PM: parabolic mirror; EOS: electro-optical sampling) About the sample: GaP and diamond wafers are used to track the THz electric field waveform and calibrate the THz electric field strength; water, alcohol and diamond are used in a series of TKE comparative experiments.

The full complex response function $R(\omega)$ of the detector is given by:

$$R(\omega) = G(\omega) \cdot A_{tr}(\omega) \cdot r_{41} \tag{1}$$

where $G(\omega)$ is a complex function for correcting the mismatch between the THz phase velocity and the group velocity of the optical pulse.

$$G(\omega) = \frac{c \times [\exp\{-2i\pi\omega d(n_g(\lambda_0) - n(\omega))/c\} - 1]}{-2i\pi\omega d[n_g(\lambda_0) - n(\omega)]} \tag{2}$$

$A_{tr}(\omega)$ is the frequency-dependent amplitude transmission coefficient due to the reflection loss at the detector surface.

$$A_{tr}(\omega) = 2/[n(\omega)+1] \tag{3}$$

$r_{41}(\omega)$ is the electro-optic coefficient of the sensor material affected by dispersion and resonant enhancement.

$$r_{41}(\omega) = r_e \times \left[1 + C\left(1 - \frac{(\hbar\omega)^2 - i\hbar^2\omega\gamma}{(\hbar\omega_{TO})^2}\right)^{-1}\right] \tag{4}$$

where $n_g(\lambda_0)$, $n(\omega)$ represent the group refractive index of the optical pulse and the complex refractive index of the THz radiation, respectively.

According to the full complex response function $R(\omega)$ of the GaP crystal, the THz time and frequency domain signals are reconstructed, and the results are depicted in Fig. S2.

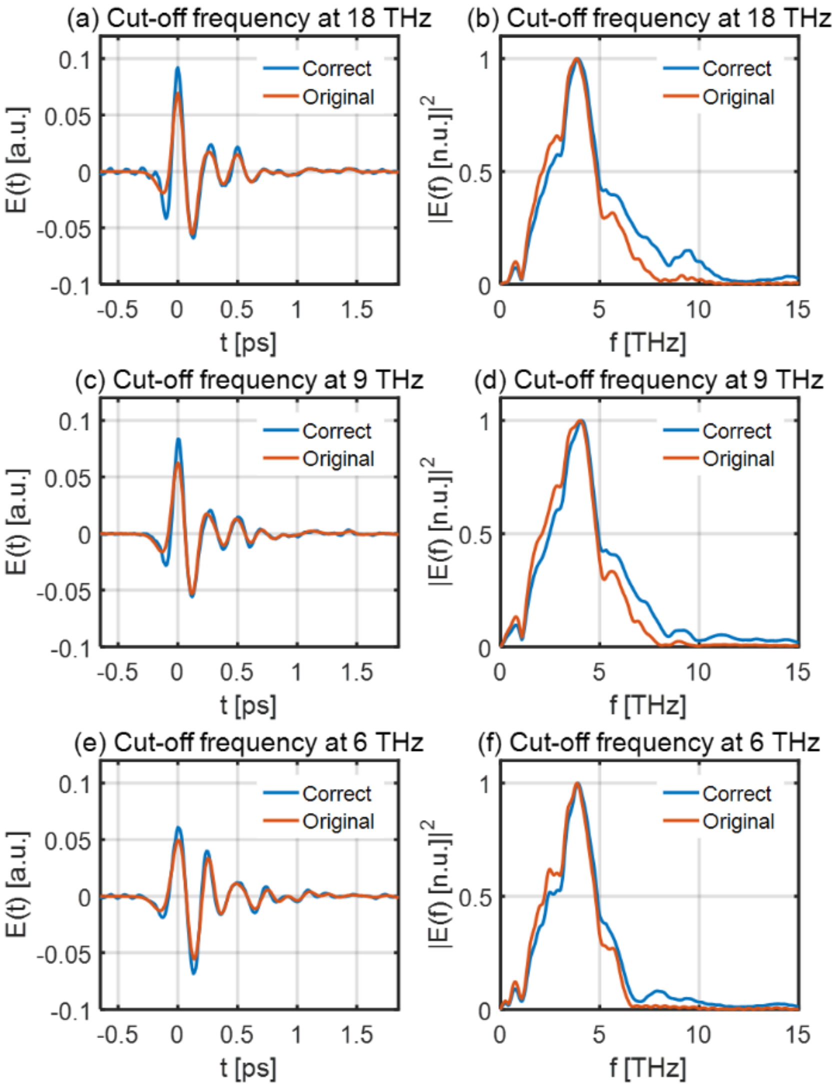

Fig. S2 a Time-domain waveform and **b** corresponding frequency-domain information of the THz electric field generated from DAST with 18 THz low pass filter (LPF). The experimental data and the corrected results are shown with red and blue lines, respectively. Similarly, we also measure the THz time-domain waveforms and corresponding frequency-domain information with **c-d** 9 THz and **e-f** 6 THz LPFs and calibrate the experimental results.

SII. Electric field strength dependence of the THz Kerr signal

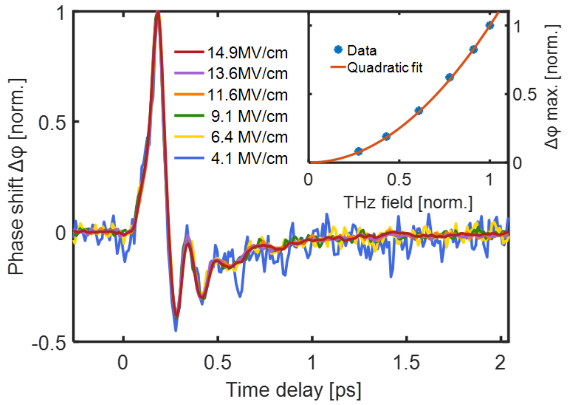

Fig. S3 THz-induced Kerr effect with different input pump electric fields in water. We normalize all the curves, and the inset shows that all the features of the responses scale with the square of the THz electric field.

SIII. The frequency dependent TKE experiments of heavy water

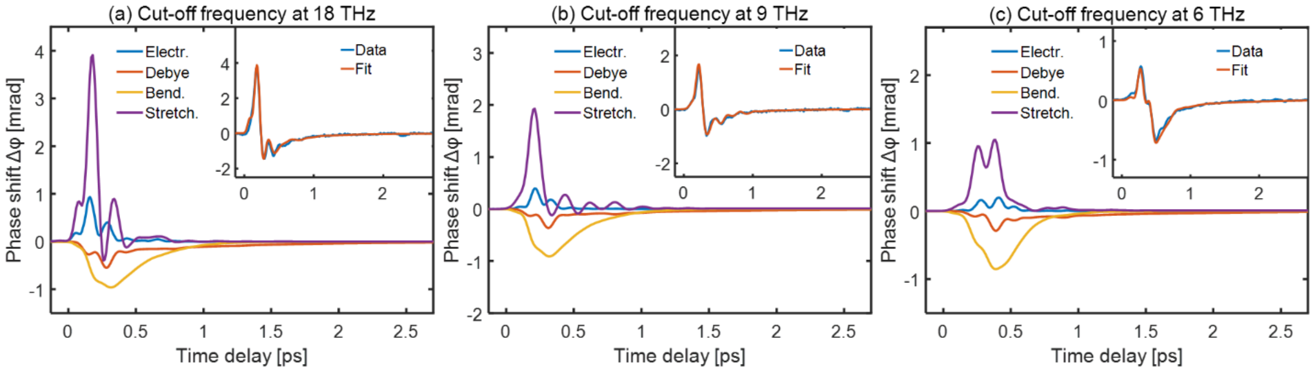

Fig. S4 Theoretical simulation of the TKE response of heavy water for the electronic, Debye relaxation, hydrogen-bond stretching vibration and hydrogen-bond bending vibration contributions under different pump electric fields with cut-off frequencies of **a** 18 THz, **b** 9 THz, and **c** 6 THz. The corresponding inset shows a comparison between the sum of all the contributions and the measured data.

SIV. The Gouy-phase shift has no effect in our experiment

For THz pump pulses of different frequencies, the spot sizes, Rayleigh lengths, and the positions of the beam waist will change slightly. The potential Gouy-phase shift may affect the actual THz electric field waveform. However, in our experiments, the effect of the Gouy-phase shift is negligible[8].

We define the collinear propagation direction of the THz wave and 800 nm beam as the Z-axis. The independent variable z is the position of the detector (GaP crystal or water film) in the experimental system, and z_0 represents the center of the THz beam waist. The positions of GaP crystal or water film are scanned along Z-axis, and the peak points of time-domain waveforms corresponding to the detector positions are plotted in Fig. S5.

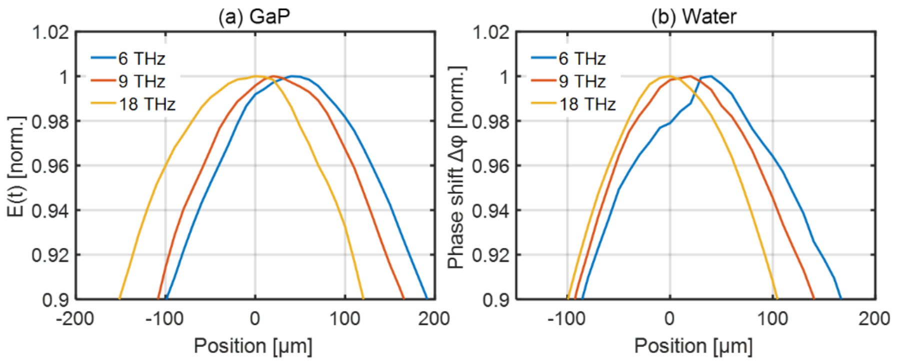

Fig. S5 The peak points of time-domain signals corresponding to the **a** GaP crystal and **b** water film positions.

We define the central position of the THz beam waist under the 18 THz LPF as the zero position, that is, $z_{0(18T)} = 0$. Therefore, the slippages of beam waist under different LPFs are $z_{0(9T)} \approx 20\,\mu\mathrm{m}$, $z_{0(6T)} \approx 40\,\mu\mathrm{m}$, respectively. In this experimental system, the diameter of THz beam is about 3 cm, and the focal length of the parabolic mirror is 5.08 cm. It can be estimated that for a THz pump pulse with a center frequency of 3.9 THz, the Rayleigh length is about 0.26 mm. Therefore, the Gouy-phase shifts caused by switching different LPFs are $\Phi_{G(9T)} \approx 0.0244\pi$, $\Phi_{G(6T)} \approx 0.0486\pi$, respectively.

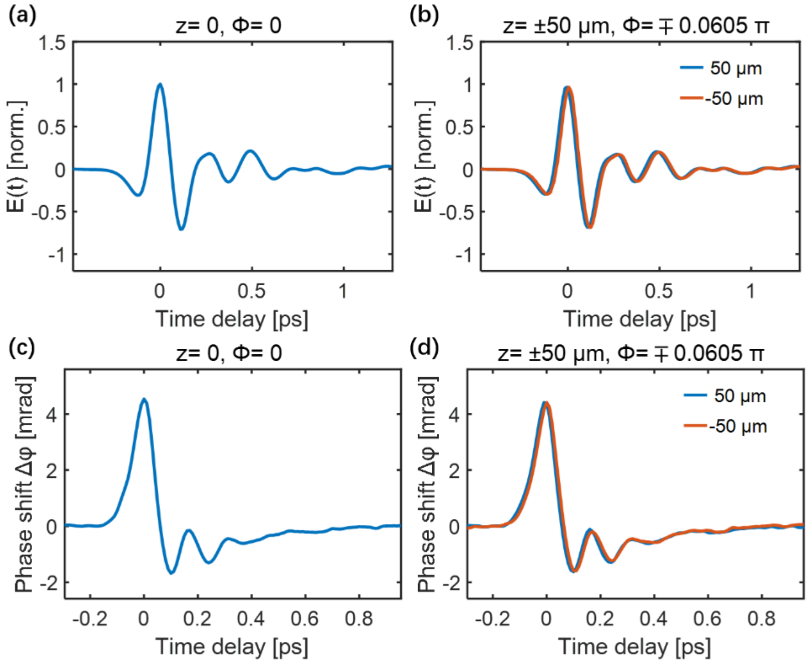

Fig. S6 The THz electric field waveform under 18 THz LPF with the GaP crystal position of **a** z=0, **b** z=±50 μm. The TKE response of water under 18 THz LPF with the water film position of **c** z=0, **d** z=±50 μm.

We measure the transient birefringence response of the detector position at $z = 0$ and $z = \pm$ 50 μm (Fig. S6). When the detector positions are ± 50 μm (the corresponding Gouy-phase shifts are $\mp 0.0605\pi$), the transient birefringence response of water film driven by a THz pulse does not change significantly. Since the maximum phase shift ($\Phi_{G(6T)} \approx 0.0486\pi$) is less than 0.0605π, the Gouy phase shift caused by switching the LPFs can be ignored.

SV. Removal of the background response

In the experiment, the system was continuously purged with dry nitrogen gas to eliminate the absorption of water vapor and the gas pressure in the box is constant. In addition, we improved the experimental device to minimize the influence of water vapor on the measurement signal. We adopted the method commonly used by previous researchers to effectively reduce water vapor diffusion[9]. In this method, the water is transported to the reservoir through a funnel and a silicone tube, and the reservoir is placed outside the inflatable box to reduce the absorption of THz wave caused by water evaporation. The method can ensure that water vapor does not accumulate around the water film, which effectively suppresses the interaction between water vapor and the THz electric field. The previous THz spectroscopic results have demonstrated the effectiveness of this method[9].

However, the nitrogen gas has a small TKE response in our experimental condition. Fig. S7a shows the TKE background responses of nitrogen gas with different THz filters (remove the water film). From the figure, we can observe N_2 revivals[10]. These results show that nitrogen gas is likely to affect the TKE response of water. In fact, we observed the revival characteristics of nitrogen gas from the raw data of the TKE response of water (Fig. S7b). We believe that the fluctuations of the TKE signal at about 2 ps, 4 ps come from the N_2 revivals. In addition, we did not observe the TKE response characteristics of water vapor (positive response with a relaxation curve extending over hundreds of picoseconds)[11], which is mainly due to the weak water vapor density and high THz driving frequency in our experiment.

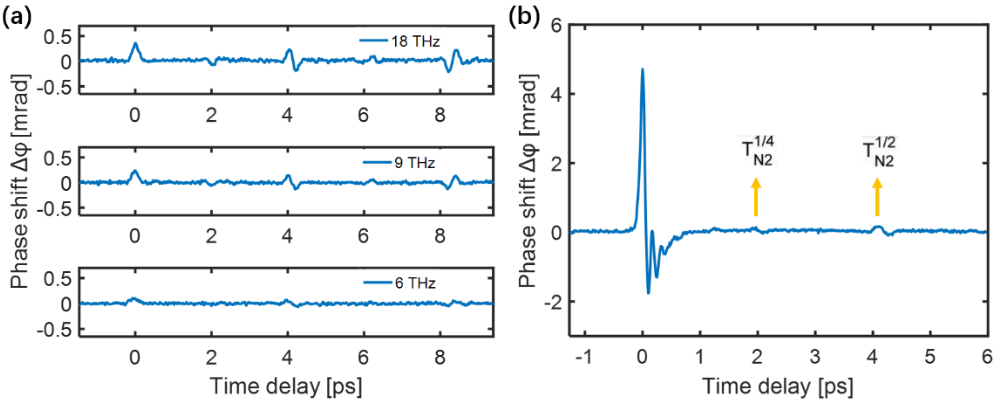

Fig. S7 a The TKE background responses with different THz filters in nitrogen gas environment. **b** Raw data for measured TKE response of water in the nitrogen gas environment with a cut-off frequency of 18 THz.

Since the water film occupies the focal position of THz wave and water has a strong absorption coefficient in the THz band, the TKE response of nitrogen gas will be reduced due to the presence of water film. We reduced the TKE response of nitrogen gas by a factor of about 1.5 to obtain the red line shown in Fig. S8a. This red line is consistent with the nitrogen gas portion of the raw data for the TKE response of liquid water (blue line shown in Fig. S8a). Therefore, the actual TKE response of water (Fig. S8b) is the result of the difference between the two lines (blue and red lines in Fig. S8a).

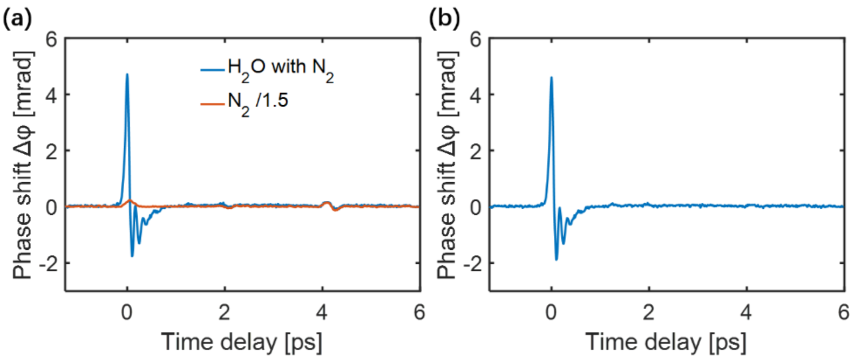

Fig. S8 a The TKE response of water film (blue) and nitrogen gas (red) under nitrogen gas environment with a cut-off frequency of 18 THz. **b** The actual TKE response of water, which is obtained by subtracting the red line from the blue line in Fig. S8a.

With the above method, the data shown in our manuscript have all been subtracted the contribution of nitrogen gas to TKE signal.

SVI. Analysis of heating effects

In this work, the pulse fluence at the center of the beam waist is about 30 mJ/cm^2. Considering the factors such as the heat capacity of liquid water, the reflectivity of the front surface of water, the THz wave absorption of water (We estimate the effective medium length of THz wave in water is about 25 μm in our experiment. At this distance, 90% of THz energy is absorbed by water.), and assuming that all the heat is transferred to the excited volume of water instantaneously without dissipation, each THz pulse can increase the water temperature by approximately 2.6 °C. However, this heating process requires a certain time $\tau = r^2 / D$ [12-14], where r is the characteristic length and D is the thermal diffusivity ($\sim 1.47 \cdot 10^{-7} m^2 / s$ in water). If we take for simplicity a sphere with the radius of about 49 μm equal to the excited volume, we get $\tau \approx 16\ ms$. In our experiment, the THz pulse duration is far less than the thermalization time, thus we believe that the heating effect within extremely short pulse duration is negligible.

In addition, the stable gravity-driven, free-flowing water film used in our experiment has the flow rate of 10 mL min^{-1}. Within the time interval of 1 ms between two adjacent pulses, the distance that the gravity-driven water film will fall is about ~ 370 μm, which is much larger than the pump spot size of 160 μm. Therefore, there is no heat accumulation between two adjacent pulses.

Moreover, it is challenging to analyze the time-dependent temperature disturbance in the sample excited by an ultrashort pulse until now. In general, this thermal disturbance has a decay time of more than 20 ps in liquid water[14-16]. However, in our work, the measured TKE signal of water decreases to the noise level after ~ 1.5 ps upon THz pulse excitation. The experimental results do not reflect the signal characteristics caused by the non-diffusive thermalization effects.

In summary, we believe that the contribution of heating effects to the TKE signal in the experiment is negligible.

References

1. Sajadi, M., Wolf, M. & Kampfrath, T. Terahertz-field-induced optical birefringence in common window and substrate materials. *Opt. Express* **23**, 28985-28992 (2015).
2. Cornet, M., Degert, J., Abraham, E. & Freysz, E. Terahertz Kerr effect in gallium phosphide crystal. *J. Opt. Soc. Am. B* **31**, 1648-1652 (2014).

3. Gallot, G. & Grischkowsky, D. Electro-optic detection of terahertz radiation. *J. Opt. Soc. Am. B* **16**, 1204-1212 (1999).
4. Leitenstorfer, A., Hunsche, S., Shah, J., Nuss, M. C. & Knox, W. H. Detectors and sources for ultrabroadband electro-optic sampling: Experiment and theory. *Appl. Phys. Lett.* **74**, 1516-1518 (1999).
5. Wu, Q. & Zhang, X. C. 7 terahertz broadband GaP electro-optic sensor. *Appl. Phys. Lett.* **70**, 1784-1786 (1997).
6. Bakker, H. J., Cho, G. C., Kurz, H., Wu, Q. & Zhang, X.-C. Distortion of terahertz pulses in electro-optic sampling. *J. Opt. Soc. Am. B* **15**, 1795-1801 (1998).
7. Casalbuoni, S. et al. Numerical studies on the electro-optic detection of femtosecond electron bunches. *Phys. Rev. ST Accel. Beams* **11**, 072802 (2008).
8. Ahmed, S., Savolainen, J. & Hamm, P. The effect of the Gouy phase in optical-pump-THz-probe spectroscopy. *Opt. Express* **22**, 4256-4266 (2014).
9. Kondoh, M. & Tsubouchi, M. Liquid-sheet jets for terahertz spectroscopy. *Opt. Express* **22**, 14135-14147 (2014).
10. Shalaby, M. & Hauri, C. P. Air nonlinear dynamics initiated by ultra-intense lambda-cubic terahertz pulses. *Appl. Phys. Lett.* **106**, 181108 (2015).
11. Kampfrath, T., Wolf, M. & Sajadi, M. The sign of the polarizability anisotropy of polar molecules is obtained from the terahertz Kerr effect. *Chem. Phys. Lett.* **692**, 319-323 (2018).
12. Qin, Z. & Bischof, J. C. Thermophysical and biological responses of gold nanoparticle laser heating. *Chem. Soc. Rev.* **41**, 1191–1217 (2012).
13. Novelli, F., Chon, J. W. M. & Davis, J. A. Terahertz thermometry of gold nanospheres in water. *Opt. Lett.* **41**, 5801 (2016).
14. Novelli, F. et al. Strong Anisotropy in Liquid Water upon Librational Excitation using Terahertz Laser Fields. Preprint at https://arxiv.org/abs/1809.04261 (2018).
15. Lock, A. J., Woutersen, S. & Bakker, H. J. Ultrafast Energy Equilibration in Hydrogen-Bonded Liquids. *J. Phys. Chem. A* **105**, 1238–1243 (2001).
16. De Marco, L., Fournier, J. A., Thämer, M., Carpenter, W. & Tokmakoff, A. Anharmonic exciton dynamics and energy dissipation in liquid water from two-dimensional infrared spectroscopy. *J. Chem. Phys.* **145**, 094501 (2016).
